# The pathway to consultation for rheumatoid arthritis: exploring anticipated actions between the onset of symptoms and face-to-face encounter with a healthcare professional

**DOI:** 10.1186/s12891-017-1619-9

**Published:** 2017-06-14

**Authors:** Gwenda Simons, Sophie Lumley, Marie Falahee, Kanta Kumar, Christian D. Mallen, Rebecca J. Stack, Karim Raza

**Affiliations:** 1 Rheumatology Research Group, Institute of Inflammation and Ageing, College of Medical and Dental Sciences, The University of Birmingham, Birmingham, UK; 2 Faculty of Medical and Human Sciences, School of Nursing, University of Manchester, Manchester, UK; 3 Arthritis Research UK Primary Care Centre, Research Institute for Primary Care & Health Sciences, Keele University, Keele, UK; 4 College of Business Law & Social Sciences, School of Social Sciences, Nottingham Trent University, Nottingham, UK; Rheumatology Research Group, Institute of Inflammation and Ageing, College of Medical and Dental Sciences, The University of Birmingham, Birmingham, UK; 5 Institute of Inflammation and Ageing, College of Medical and Dental Sciences, The University of Birmingham, Birmingham, UK; Sandwell and West Birmingham Hospitals National Health Service (NHS) Trust, Birmingham, UK

**Keywords:** Arthritis, rheumatoid, Help-seeking behaviour, Delayed diagnosis, Information seeking behaviour, Self-management of symptoms

## Abstract

**Background:**

When people first experience symptoms of rheumatoid arthritis (RA) they often delay seeking medical attention resulting in delayed diagnosis and treatment. This research assesses behaviours people might engage in prior to, or instead of, seeking medical attention and compares these with behaviours related to illnesses which are better publicised.

**Methods:**

Thirty-one qualitative interviews with members of the general public explored intended actions in relation to two hypothetical RA vignettes (with and without joint swelling) and two non-RA vignettes (bowel cancer and angina). The interviews were audio-recorded and transcribed. Analysis focused on intended information gathering and other self-management behaviours in the interval between symptom onset and help-seeking.

**Results:**

Participants were more likely to envision self-managing symptoms when confronted with the symptoms of RA compared to the other vignettes. Participants would look for information to share responsibility for decision making and get advice and reassurance. Others saw no need for information seeking, perceived the information available as untrustworthy or, particularly in the case of bowel cancer and angina, would not want to delay seeking medical attention. Participants further anticipated choosing not to self-manage the symptoms; actively monitoring the symptoms (angina/ bowel cancer) or engaging in self-treatment of symptom(s).

**Discussion:**

These results help define targets for interventions to increase appropriate help-seeking behaviour for people experiencing the initial symptoms of RA, such as educational interventions directed at allied healthcare professionals from whom new patients may seek information on self-management techniques, or the development of authoritative and accessible informational resources for the general public.

## Background

Early diagnosis of rheumatoid arthritis (RA) is associated with early intervention and improved long-term prognosis [1]. However, for a variety of reasons, including symptom recognition, misperceptions about causality and the long term consequences of symptoms [2–6], patients often delay presenting to their general practitioner (GP) or family physician [7, 8] resulting in a potential delay in diagnosis and the start of treatment. Delayed treatment of RA increases the risk of joint damage and disability [1, 9].

The ‘Model of Pathways to Treatments’ [10, 11] describes events, processes and intervals that may occur in the period prior to the start of treatment and provides a helpful framework to identify where delay may occur in a patient’s journey. The main events in the model are: Detection of bodily changes; Perception of reason to discuss symptom with healthcare professional; First consultation with healthcare professional; Diagnosis; and Start of treatment. The model further describes the intervals between those events and processes within those intervals. It also identifies the factors that might influence how long an interval might last. For the current paper we focus on the so-called ‘appraisal interval’ which refers to the time between the detection of bodily changes and perceiving a reason to contact a health care professional [10, 11]. There are a number of actions people can take in this interval which may in fact delay them seeking medical attention. For example, there is evidence that patients often normalise symptoms, self-medicate with analgesics, or modify behaviours to offset the impact of the symptoms [12–14] before or instead of consulting a health care professional.

Before deciding to consult a health care professional, patients might further choose to seek information about their symptoms, either through discussion with others (e.g. family members [15]) or by consulting written resources (e.g. leaflets). Patients are also increasingly using the internet as a resource for health information [16–18] including information about RA [19].This information seeking might be conducted for various reasons, for example to find the possible cause of the symptoms or to inform decision making about whether or not to seek medical attention. Depending on the quality or nature of the information provided this may encourage or delay people from seeking medical attention [3]. Both types of actions (information seeking and self-management) map onto ‘patient appraisal and self-management’ processes within the ‘Appraisal interval’ in the model of pathways to treatment [10, 11].

We currently do not have sufficient understanding of the full spectrum of actions people may take if they were to develop symptoms of RA; where and how they might search for information and how they might (or might not) attempt to manage their symptoms before seeking medical help. As a result potentially important opportunities for targeting interventions are missed.

Chest pain and rectal bleeding are also indicative of potentially serious underlying conditions (e.g. ischaemic heart disease and bowel cancer). In a recent mixed method paper we explored the proposed speed with which members of the public would seek help for the symptoms of angina, bowel cancer and RA and found that respondents intended to seek medical attention significantly more quickly for bowel cancer and ischaemic heart disease symptoms than they would for any given combinations of RA symptoms. Accurate symptom recognition and the perception that symptoms are indicative of a serious underlying condition were both found to be important drivers for rapid help-seeking and more likely to be established for bowel cancer and angina then for RA. [6] Symptoms of bowel cancer and ischaemic heart disease feature commonly in the media and in public health campaigns and research suggests this is associated with faster consultation in the UK [20, 21]. Such messages are much less apparent for RA.

A better understanding of the actions of people within the appraisal interval for both well published diseases as well as RA may provide further targets for intervention to reduce delay in help-seeking for symptoms of RA. In a secondary analysis of the interview data reported in our mixed method paper [6], the current paper addresses the proposed actions of participants in the period between first noticing the symptoms and seeking medical attention in response to the hypothetical onset of typical symptoms of bowel cancer and angina compared with responses to typical RA symptoms.

## Methods

### Participant inclusion criteria and recruitment

Participants for this study were recruited through 2 inner-city GP practices and through posters displayed at Keele and Birmingham University. Full details are presented elsewhere [6]. Briefly, names of potential participants were extracted from the patient list of participating GP practices, excluding persons with a diagnosis of inflammatory arthritis. Participants were purposively sampled from three age groups (18–40, 41–60 and over 61 years) with the largest group being the 41–60s. This allowed the final sample to demographically reflect the age distribution for RA onset. The extracted lists were screened by a GP to exclude vulnerable persons who were deemed unsuitable for participation in the study. Suitable potential participants were sent an invitation and those individuals wishing to take part contacted the researcher to arrange an interview. The current article covers a secondary analysis of the interview data looking specifically at the actions participants proposed to take in the appraisal interval, however long or short this might be. The original analysis focused on the role of symptom recognition in help-seeking for the symptoms of RA and compared this with help-seeking for symptoms of angina and bowel cancer [6].

### Data collection and analysis

The semi-structured interviews were recorded and transcribed verbatim. During the interview participants were presented with four symptom vignettes (1. Inflammatory joint symptoms: peripheral joint pain and morning stiffness; 2. Typical early RA: peripheral joint pain, morning stiffness and swelling of the joints; 3. Bowel cancer: per-rectal bleeding and change in bowel habit and 4. Angina: chest pain on exertion; See Table 1 for sample vignettes).

**Table 1:** Sample vignettes

RA with pain, stiffness and swelling
Increasingly you find it difficult to get out of bed in the mornings and move around because your joints are very stiff. In particular your finger joints and wrist joint are painful and they hurt most when you wake in the morning, but the pain can last all day. As a consequence of the stiffness you take longer than usual to get out of bed, and it can take some time before the stiffness subsides, and you are able to move around without much difficulty. You look at your wrist, knees, and hands and you notice that some of your joints are swollen and are sensitive to touch. You have not experienced these symptoms before.
Bowel cancer
You visit the bathroom and notice that there is blood in your stools. This has happened several times over the last month. Recently you’ve been going to the toilet more often and have had some diarrhoea. You have also noticed that you have been losing weight, which is unusual because your appetite has been normal and you have not been exercising more than normal. You are also feeling run down and very tired.

Participants were then asked what they would do if they experienced these symptoms (see Table 2 for an extract from the interview guidelines). Alternating participants were presented with either the RA vignettes (1.RA joint pain and stiffness; followed by 2.RA joint pain, stiffness and swelling) or the non-RA vignettes first (3.Bowel cancer; followed by 4.Angina).

**Table 2:** Extract interview guidelines

• How long would you wait before you sought medical attention with these symptoms?
• Would you want to get more information about these symptoms before you seek medical attention?
• How would you go about finding more information?
• If you were to experience these symptoms, would you discuss them with anyone other than your GP?
• What is the main reason you would discuss the symptoms (with this person/ with your partner/ with….)?
• Is there anything you think you can do yourself to alleviate these symptoms?

Transcripts were analysed thematically [22]. Blind coding of four randomly allocated full transcripts was conducted by GS and SL to ensure similar reasoning and agreement on emergent codes; no further double coding was required. Emerging themes were discussed between SL and GS and checked by KR. The themes in the current paper relate to discussions around intended information-seeking and other self-management behaviours people intended to undertake in the period prior to the first consultation with a health care professional.

## Results

A total of 32 people were recruited into the study (31 by post and 1 through poster advertisement). The data from the only telephone interview had to be discarded due to technical problems. Thirty of the 31 remaining participants were interviewed at their GP practice and 1 at the University of Birmingham. Participants (16 females) were aged between 23 and 84 years (*Mean* = 63) and all but two were of a White British background. Almost half of the participants (*n* = 14) were in work at the time of the interview; the others were either retired (*n* = 15), in education (*n* = 1) or looking for work (*n* = 1). Seven participants indicated that they knew someone with RA, 9 knew someone with bowel cancer, and 9 knew someone with angina. Twenty-four described having joint problems themselves, 4 had had some form of cancer, 3 indicated having heart problems and 15 reported other complaints. Six participants had a (para)medical background (e.g. former nurse).

The actions participants proposed to engage in before consultation when confronted with the various symptom vignettes were divided into two overarching themes: (1) seeking information about the symptoms, and (2) the self-management of the symptoms (or the lack thereof). Both the themes and subthemes are described below with illustrative quotes in Tables 3 and 4.

**Table 3:** Quotes related to Information-seeking

Quote number	Quote (participant number/ disease referred to)
Why would participants discuss symptoms with others?	
1	I’ve got a GP friend at Shul. I suppose if I was a bit, you know, a bit worried about something, I would ask him. (p20/bowel cancer)
2	Well, I’d ask my wife and she actually mixes with a lot of pharmacists. One or two of them seem to be quite level headed, if you’ll excuse me saying that. (p08/angina)
3	Yes, if there was friends and family that I knew were in the same situation. Yeah, I think swapping advice and, you know, how they cope with it does help… (p19/RA with swelling)
4	[I would talk to] My husband…So and, that’s, as I said, is that because sort of try and determine whether or not to go or … (p24/angina)
5	Yeah, I’d talk - I talk to my wife every time. I mean, she’s my partner …there’s no secrets there. (p12/angina)
6	I would I mean obviously people that I trust, my family members and anybody that might have…(p05/ bowel cancer)
7	I would discuss it with my husband yeah – yes we’ve got quite a good relationship you know so there’s not a lot that we don’t know about each other’s health and routines and things like that. (p14/bowel cancer)
8	We have a gathering Wednesday nights and Friday afternoons. We have a game of bingo. So that’s when you-in between we have a cup of tea and a chat you know. That’s when it would all come up. (p31/RA with swelling)
9	Oh this, this happened to me. Have you ever had that?’ you know you just talk about things like that don’t you, really. (p15/angina)
10	Talk this through with somebody, is it me just going off on one and making this a bigger thing than it is. And sometimes maybe an objective opinion in something like that would be… but I’d want to ring someone who wouldn’t overreact. (p16/angina)
11	And almost leave other people to make the decision, or make the decision together. (p25/angina)
12	I would definitely mention it to someone, that I didn’t feel quite right, and, ‘I’m gonna have a cup of tea,’ and where I’m going, just in case I collapse or something. (p30/angina)
Why would participants not discuss symptoms with others?	
13	No, I don’t think so. If I wasn’t worried myself, then I wouldn’t. (p31/angina)
14	I’ve got quite a bit of medical knowledge, so I’ve got a good idea of what, you know, what things are. (p20/bowel cancer)
15	Well I wouldn’t tell my daughter, I wouldn’t worry her and no I don’t think so, I think initially it would just be my husband. (p03/angina)
16	That’s what I miss and I think the older you get, the more you miss that because it is more difficult to, you know.. I mean, the neighbours are very good and I meet people socially at the U3A, but not people you could really talk to and I think that is to do with age (p28/angina)
17	…But I’d be a bit more embarrassed than with the other symptoms… I don’t know; when something’s toilet related you just don’t wanna talk about it as much. (p26/bowel cancer)
18	No, this is a bit vague, really, to get – I think I’d want to go in straight to the GP [General Health Practitioner], there and tell him exactly what’s been going on… (p04/angina)
Why would participants seek information from written sources or the internet?	
19	I’m not brilliant with the computer, but I just tend to go onto Google and then type in what it is that I’m looking for (p19/RA)
20	I’d look at authoritative ones because I think there are a lot of, so I’d be looking for ones like the Boots site, Boots have one or NHS Direct or official sites. At this point I wouldn’t looking for sort of anecdotal sites like forums you know where people are sharing experiences because it just tends to frighten you quite frankly when you look at those things so I’d be looking for informed opinion and – and factual opinions rather than, you know rather than personal feelings (p07/RA with swelling)
21	Mmm…I suppose the only thing with this is that I’d want to know if it was a progression of my own illness, so that I could rule that out. [I: … or how would you find out whether it is a progression of your own illness?] Initially the internet and databases and things just to look at… just to rule that out. (p16/RA)
22	I get all the information from the various magazines form the Parkinson’s association. (P18/bowel cancer)
23	Yeah I think with the internet if you think you know what it is you might sort of go and look on it because then you check, you know check your self-diagnosis and you check and you think oh yes I’ve got that and that and that, oh it sounds like that’s what it is right well I’ll just go and sort of see the doctor. Whereas if it’s sort of, you’ve got hundreds of, not hundreds but a number of different things and you’re not really sure then I’d just sort of […] let the expert decide I think. (p14/ RA with swelling)
24	Yes, I’d need more information before I went to the doctor, so I could understand more about it and I could explain to him what’s happening in more detail. (p18/ bowel cancer)
Why would participants not seek information from written sources or the internet?	
25	I know people do look on the internet and things, but I don’t really – every single symptom seems to lead to cancer on the internet and so I don’t really go there that much. (p26/RA)
26	I have a lack of trust of stuff on the internet, so…yeah. (p26/RA)
27	If you start self-diagnosing, and you start cross reading things, you’ll make yourself even worse; you’ll make yourself panic (p12/angina)
28	My experience of the internet is, it will answer your questions but you have to know the question to ask. If you are too vague, you get a whole load of information which, you know, whether it’s my age, but the internet is not the first point of call (p15/RA)
29	I just wouldn’t - you end up confusing yourself on the whole, so it’s far better, once it gets painful enough and I’m in a mess as it were, or whatever, there’s only one place, the doctor (p13/RA)
30	Now if he said yes it is cancer then I’d be looking at, on the internet about well what’s good to eat and how can you change your lifestyle if you’ve got cancer but I wouldn’t … (p07/bowel cancer)
31	I haven’t got a computer at home, so I don’t know how to use it. (p18/bowel cancer)

**Table 4:** - Quotes related to Management of symptoms

Quote number	Quote (participant no/disease quote refers to)
No self-management of symptoms	
1	Well, I’d very likely put up with it for a while, you know, do - rub it, or whatever. Pink, you know, it’s - I’ll let it go for a while (p31/RA with swelling)
2	I mean if your joints are swollen and sensitive I don’t know what you can do (p24 /RA with swelling)
3	I think if it was blood, I would go and see the doctor, because there’s nothing you can do about that (p20/bowel cancer)
4	I mean there’s not really any way that you could take any sort of medication for anything (p12/angina)
5	I wouldn’t even contemplate doing anything myself I would just go to the doctors immediately, as soon as I saw blood in my stool I wouldn’t even contemplate any sort of preventative or – or you know any action to make it better I would just go and see the doctor straight away (p07/bowel cancer)
Active monitoring	
7	I’d want to monitor that a bit. It depends really on what sort of heaviness and what sort of tightness, if it was particularly tight (p16/angina)
Manage, treat or prevent the symptom(s).	
8	Well, I’d then likely take some paracetamol … stretching and, manipulating … trying some kind of soaking in warm water perhaps or anti-inflammatory gels, rubs, that sort of thing (p01/RA)
9	I think people say, you know, move them, move your body, you know, don’t be lazy. The more you move and do exercises, it’s all part of whole (p02/angina)
10	I wouldn’t even contemplate doing anything myself I would just go to the doctors immediately, as soon as I saw blood in my stool I wouldn’t even contemplate any sort of preventative or – or you know any action to make it better I would just go and see the doctor straight away (p07/bowel cancer)
11	If it’s a pain in the chest, … I keep some …Gaviscon, is it? (p31/angina)
12	Well I think you’ve got to watch your diet and your lifestyle … if you know you’re sort of tending to drink a bit heavily you’d sort of think well I’d better moderate this or smoking is the prime example, if you were a smoker you should give up definitely and diet you know you’ve got to sort of watch what you eat, make sure that you eat sort of healthily (p14/angina)
13	If it’s something which I can help myself – take things a bit easier, you know, do – if you sort of – if you do strenuous activities then you experience more problems, which I think as you get older you do, then you stop trying to do such strenuous things (p28/angina)
14	Take my inhalers, sit down and relax. Check if that works (p26/angina)

### Seeking information about the symptoms

Participants identified a number of sources of information they would access for further information about the symptoms they were presented with, including people such as friends, family, colleagues, and non-medical healthcare professionals (e.g. pharmacists or physiotherapists), written materials such as books, magazines, information leaflets and the internet. The subthemes extracted relate to the reasons why participants thought they would or would not utilise these sources of information.

*Why* ***would*** participants discuss symptoms with others? The most common reason for intending to go to a certain person for information was that this person was someone they perceived as having a level of expertise on the subject (Table 3, Quote 1: T3Q1). This could take the form of either perceived medical expertise (such as a pharmacist; T3Q2) or experiential expertise due to the person(s) experiencing similar symptoms (T3Q3). Participants often indicated that they would be seeking advice about what they should do about the symptoms or what the cause might be (T3Q4).

Participants further expressed the intention to discuss their symptoms with the person they usually discussed important things with, such as their partner or children (T3Q5). This related to the high level of trust in this person’s opinion (T3Q6), and the fact that this person knew them and their previous history (T3Q7). Sometimes, participants intended to discuss their symptoms with a ‘convenient’ person. For example, someone they see regularly through involvement in a shared activity such as a weekly yoga class or coffee morning (T3Q8).

When contacting non-medical friends and family, many participants indicated that they would not necessarily be looking for more information about the symptoms or suggestions for a specific diagnosis, rather they would want to share the burden of decision making regarding the symptoms. In particular, they would be looking for reassurance that their chosen course of action was appropriate or that the symptoms were not indicative of a serious underlying condition (T3Q9). This reason was given for the discussion of symptoms with others in reaction to all 4 vignettes. Participants further indicated wanting to either get a second opinion on their own reasoning about the symptoms (T3Q10) or to share the responsibility of the decision making (T3Q11). Further, in response to the angina vignette, participants stated they wanted someone to know about their symptoms so that they might take action if something happened acutely (T3Q12).

*Why would participants* ***not*** *discuss symptoms with others?* Some participants indicated that they would not discuss their symptoms with other people, for example if they were not particularly worried about the symptoms (T3Q13). Some participants (mostly those with a medical or related background), felt that they would not need to ask people for advice or information because their own knowledge was sufficient (T3Q14). Others indicated that they would not want to worry those close to them (T3Q15). Another reason stated by interviewees was being socially isolated and thus not having anyone to talk to about the symptoms (T3Q16). Participants also indicated that they might not discuss certain symptoms due to embarrassment - this was most apparent in the bowel cancer vignette (T3Q17). Finally, some participants clearly stated they would not discuss their symptoms with others because they felt it would delay medical attention which they felt was needed imminently (T3Q18). This attitude was clearly more prevalent with regards to the angina and bowel cancer vignettes.

*Why* ***would*** *participants seek information from written sources or the internet?* In general there were more positive attitudes and general willingness to search for information through written materials or internet searches about the symptoms of RA compared to the symptoms of bowel cancer or angina. Some participants mentioned adopting very generic internet search strategies; for example entering their symptoms into a search engine and seeing what came up, or looking up ways to self-manage certain symptoms (T3Q19). Others intended to adopt a more specific on-line search strategy, for example searching particular diagnoses that they thought they had (mostly ‘arthritis’), or only looking on particular ‘legitimate’ sites, such as the National Health Service (NHS) website, for information (T3Q20). Some stated that they would specifically check to see whether their new symptoms were related to a pre-existing condition (T3Q21), they may also look through written material given to them in relation to a prior diagnosis (T3Q22).

However, for all conditions, seeking out written information was often with the proviso that if they were already worried about the symptoms they would seek medical help anyway (T3Q23). Some even stated that the purpose of searching for more information would be to build a more comprehensive picture of their symptoms and to present the doctor with better information (T3Q24).

*Why would people* ***not*** *seek information from written sources or the internet?* The main reason participants gave for intending not to search for further information, particularly from the internet, was that the information provided could be “scary” (e.g. suggesting diagnoses such as cancer or a heart attack (T3Q25)), inaccurate or untrustworthy (T3Q26). Participants were worried that the information may make them “panic” (T3Q27) or that they might misinterpret the information they obtained or receive an overload of information (T3Q28). Various interviewees indicated that they intended to wait to discuss their situation with a health professional (T3Q29) and some participants would offset the fear of searching for information on the internet by only searching a diagnosis once the diagnosis had been confirmed by a health professional (T3Q30). This strategy was much more prevalent in the non-RA vignettes. Finally, a small number of participants indicated that they did not intend to seek information from the internet because they did not have easy access to online material (T3Q31).

### Symptom-management

Subthemes related to other self-management techniques mainly involved no self-management of the symptoms; active monitoring of symptoms; and managing the symptoms themselves either as a definitive action or as an interim measure, for example whilst waiting for consultation.

#### No self-management of symptoms

Some participants indicated they would not manage their symptoms because they did not feel the symptoms were serious enough to warrant any action at all. Simply putting up with the symptoms or taking some marginally action was exclusively mentioned in response to the two RA vignettes (T4Q1). Others were not sure what they could actually do to improve the symptom(s). This was a reason stated in particular in relation to RA with joint swelling (T4Q2) and bowel cancer symptoms (T4Q3). Participants also had problems envisioning what they could do to manage the symptoms of angina (T4Q4). Finally, some participants intended to seek medical attention straight away obviating the need to self-manage their symptom(s) (T4Q5). This was usually in response to the angina and bowel cancer vignettes.

#### Active monitoring

A theme emerging in particular for the non-RA vignettes was that participants would ‘actively monitor’ their symptoms, an approach that differs from simply ignoring a symptom and getting on with life (previously discussed). ‘Monitoring’ would involve a heightened awareness of symptoms, not disguising them by for example taking painkillers (T4Q6) and in some cases participants suggested that they would keep a (mental) symptom diary (T4Q7), seeking help if symptoms worsened.

#### Manage or treat the symptom(s)

Most participants indicated, either spontaneously, or when asked directly, actions they would take to manage the symptoms with which they were presented. This was particularly evident in the RA vignette without joint swelling where the principle problem was perceived to be pain and for which participants suggested use of over-the-counter analgesic or anti-inflammatory medications or topical therapies and physical approaches (e.g. exercises, and heat) (T4Q8). Similar self-management suggestions were proposed in response to the RA vignette with joint swelling. For both RA vignettes participants further considered weight loss, rest, exercise, dietary modification and doing things that were considered ‘good for your health’ (T4Q9).

In response to the bowel cancer and angina vignettes, participants were in general much more reluctant to self-manage symptoms (T4Q10). However, some did suggest symptom-modifying actions including management of indigestion (T4Q11), taking tablets for diarrhoea for the symptoms described in the bowel cancer vignette and pain control with over-the-counter medications for angina.

Lifestyle modification was also mentioned in response to the non-RA vignettes. In the bowel cancer vignette in particular, dietary modification was singled out as a good way to reduce the chance of further changes in bowel habit and rectal bleeding (T4Q12). In response to the angina vignette participants mentioned stopping smoking and eating more healthily as well as rest and relaxation (T4Q13).

Across all four vignettes, participants with pre-existing medical conditions stated they would take steps to manage that pre-existing condition. For example the use of inhalers if they attributed the chest pain described in the vignette to having an asthma attack (T4Q14).

## Discussion

The current research provides an insight into the range of activities that patients might engage with during the ‘appraisal interval’ in the Model of Pathways to Treatment [10, 11], i.e. the time between the detection of bodily changes and perceiving a reason for consultation with a health care professional. This insight is provided both for relatively well publicised diseases, such as angina and bowel cancer, and for RA. The comparative nature of this research allowed participants to compare and contrast between symptom complexes indicative of different pathologies. Analysis of the patient appraisal and self-management processes and in particular the focus on both information seeking and other self-management activities provide targets for future intervention, such as a targeted website and other public health interventions and training of allied health professionals to reduce delay in help-seeking for people self-managing symptoms of RA.

### Seeking information about the symptoms

Many interviewees indicated that they intended to talk to others about their symptoms at some stage. This was particularly so in the case of RA symptoms. Participants indicated they would speak to someone they trusted and were close to and/or to someone with perceived expertise, either medical (e.g. physiotherapists or pharmacists) or experiential.

Some of these individuals are ideally placed to provide accurate information about RA and encourage speedy help-seeking. For example, community pharmacists are often consulted about over-the-counter analgesics, prescribed medicines and other healthcare solutions and qualitative research describes pharmacists as being a source of reassurance or to seek legitimation for a visit to the GP [23]. This suggests that allied health professionals such as pharmacists can play a vital role in directing patients with the symptoms of RA towards a medical consultation rather than prolonged self-management of symptoms.

Our data further suggest that many of the conversations that participants intended to have about their RA symptoms would be with family or friends for reassurance or help with decision making. Participants also indicated they would engage with those who appeared to have (had) similar symptoms. Previous research, both in RA and cancer, has shown that depending on the quality or nature of the information provided such interactions might in fact lead to help-seeking delays [3, 24]. Educational materials and interventions to target misperceptions of RA in the general population may help ensure that those experiencing symptoms, or those they engage with, would realise that swift help-seeking would be beneficial for the symptoms of RA thus reducing the appraisal interval.

In contrast, participants were more likely to indicate that they would forgo engagement with others about the symptoms of bowel cancer or angina prior to seeking medical attention, often recognising that the symptoms warranted urgent medical attention resulting in a short appraisal interval and an intention to see the GP quickly. Some participants indicated that the embarrassment associated with the symptoms of bowel disease that would prevent them from discussing the symptoms with others and others preferred not to worry significant others with their symptoms (indicating that they associated the symptoms with a serious underlying cause).

The interview data related to searching for information in written materials or online when confronted with the symptoms of RA, angina or bowel cancer again provide useful insights for possible future interventions. Many comments specifically related to the internet. Some participants indicated that they would not consider looking up symptoms on the internet for fear of coming across ‘scary’ diagnoses. This was often mentioned in relation to symptoms related to bowel cancer, but also for the other symptom complexes.

Of those participants who would use the internet, most would only review information from websites considered legitimate, e.g. those linked to the NHS. In addition, many stated they would only look on the internet once they have some idea of a possible diagnosis, for example ‘arthritis’. However, an exploratory study assessing the accuracy of symptom checkers in the context of patients with new onset inflammatory arthritis found that the diagnosis or advice offered to patients with the symptoms of RA was frequently inappropriate [25]. Thus there may be cause to review resources such as the NHS website and ensure that generic search terms such as ‘arthritis’ and ‘pain in knee’ lead to sites which identify clearly that not all arthritis is of mechanical origin, and that symptom complexes including, for example morning stiffness, swelling and pain may be indicative of RA, which requires prompt initiation of prescribed medications. Resources related to (bowel) cancer [26] and heart disease [27] may also often be inaccurate or misleading.

### Symptom-management

Participants described a range of approaches to self-managing the symptoms of RA whereas responses to the symptoms of angina and bowel cancer were more uniform. Participants proposed to manage pain associated with RA symptoms with analgesic and anti-inflammatory medications, hot and cold compresses and lifestyle modifications. These may be appropriate ways of managing symptoms whilst waiting for a medical consultation, having made an appointment. However, many participants intended to use these varied self-management techniques for extended periods, not recognising the need for consulting a health care professional and delaying appropriate help-seeking. The failure to recognise the need for consultation might be due to misinterpretation of the symptoms or misperceptions about the seriousness of the symptoms [6, 28, 29] and is again a potential target for intervention.

In contrast, although behaviours to manage or treat symptoms were mentioned in reaction to the bowel cancer or angina vignettes, participants were unsure about the best course of action and less inclined to engage in them as an alternative to seeking prompt help from a health professional.

The subtheme of active monitoring also maps directly onto the processes described within the appraisal interval as part of the model of pathways to treatment. The principal of active monitoring, which we have interpreted as a heightened awareness of a symptom, sometimes to the extent of keeping a (mental) diary and recording changes, was hardly mentioned in relation to the symptoms of RA but was described in relation to the angina and bowel cancer vignettes. Previous qualitative research has also described a period of monitoring symptoms for exacerbations or persistence in cancer patients [24]. It is unclear whether actively monitoring the symptoms always results in speedy help-seeking. However, if people were encouraged to actively monitor their joint and related symptoms for a short period of time, this may prompt an earlier recognition of the need to seek medical help than is currently the case.

This study has a number of limitations. Firstly, responses to the hypothetical symptom vignettes may not match with actual responses were individuals to experience these symptoms. Whether behavioural intentions in a particular situation are translated into actual behaviours if the situation occurs depends on a number of factors such as having the (perceived) resources and opportunities to perform the behaviours [29, 30]. Retrospective research into help-seeking with patients newly diagnosed with a disease provides helpful insights, but such studies are limited by recall bias [2, 3, 29], which the current research design avoids. In addition, the use of hypothetical vignettes allows participants to compare their responses to symptoms across diseases), which would be difficult to achieve with a study design which involved interviewing patients post-diagnosis.

Participation in this research was self-selected. Those who chose to take part were mostly of White British origin, and as such not necessarily representative of the general population. Ethnic minority populations are underrepresented in health research generally, and efforts to increase representation should be addressed in future research [31].

## Conclusions

Our findings increase understanding of the behaviours people engage in when encountering symptoms from a variety of conditions and suggest specific opportunities for interventions to encourage appropriate and timely help-seeking for the symptoms of RA. One possible route could be to direct educational interventions at allied healthcare professionals (e.g. pharmacists) who should direct appropriate clients to consultation with their GP or other health care professional. In addition, the development of authoritative, easily identifiable and understandable information available both on the internet and as printed resources, may also improve knowledge of RA and facilitate appropriate help-seeking behaviour.

## Abbreviations

GP: General Practitioner
NHS: National Health Service
RA: Rheumatoid Arthritis
T3Q1: Table 3, Quote1
